# *In vitro* comparison of the surface roughness of three nanohybrid resin composites before and after dry and wet polishing

**DOI:** 10.4317/jced.63063

**Published:** 2025-09-01

**Authors:** Aldana Quispe-Pillco, Xiomara Bendezú-Quispe, Leonor Castro-Ramirez, Jose Huamani-Echaccaya, José Rosas-Díaz, Marysela Ladera-Castañeda, César Cayo-Rojas

**Affiliations:** 1 School of Stomatology, Universidad Privada San Juan Bautista, Lima, Peru

## Abstract

**Background:**

Nanohybrid resin composites are widely used in esthetic dentistry, and the choice of an appropriate polishing technique can influence their surface properties. The aim of this study was to compare, *in vitro*, the surface roughness of three nanohybrid resin composites before and after dry and wet polishing.

**Material and Methods:**

This *in vitro* longitudinal experimental study included 60 composite resin discs, evenly distributed into three groups (n = 20): Filtek Z250XT, Opallis, and Tetric N-Ceram. Each group was further divided into two equal subgroups (n = 10) for the application of dry and wet polishing techniques. Surface roughness was measured using a digital profilometer before and after polishing. Independent and paired Student’s t-tests were used for statistical analysis, with a significance level set at *p* < 0.05.

**Results:**

Prior to polishing, no significant differences in average surface roughness (Ra) were found among the resin groups, indicating adequate standardization: Filtek Z250XT (*p* = 0.899), Opallis (*p* = 0.585), and Tetric N-Ceram (*p* = 0.721). Following dry or wet polishing, no significant intragroup differences were observed: Filtek Z250XT (*p* = 0.066), Opallis (*p* = 0.124), and Tetric N-Ceram (*p* = 0.584). When comparing pre- and post-treatment values, Filtek Z250XT showed a significant reduction only with wet polishing (*p* = 0.003). In contrast, both Opallis and Tetric N-Ceram exhibited a significant decrease in roughness with both polishing methods: dry (*p* = 0.044 and *p* = 0.001, respectively) and wet (*p* < 0.001 for both).

**Conclusions:**

Both dry and wet polishing were effective in reducing the surface roughness of Opallis and Tetric N-Ceram resin composites, whereas in Filtek Z250XT, a significant reduction was observed only with wet polishing. These findings suggest that the effectiveness of the polishing procedure may vary depending on the type of resin composite, which should be considered when selecting clinical finishing and polishing protocols to optimize the surface properties of restorative materials.

** Key words:**Comparative study, Composite Resins, Dental materials, Dental polishing, Surface properties, surface roughness.

## Introduction

Resin composites are the restorative material of choice because they effectively mimic the tooth structure and can be used for direct restorations in both anterior and posterior teeth, offering optimal aesthetic results [1,2]. Over time, its formulation has evolved—particularly in terms of filler particle size and distribution—to enhance its physical, mechanical, and optical properties. Such improvements aim to optimize the aesthetic, biological, and functional performance of the material. In this context, nanohybrid resin composites have become the most widely used in the anterior region due to their superior characteristics [3,4]. Their composition comprises a resin matrix and both glass fillers and nanoparticles, which confer high resistance to discoloration, excellent aesthetics, and outstanding polishability. Particle sizes range from 0.02 to 1 µm, fulfilling the aesthetic requirements necessary to achieve a high level of gloss and surface finish [5-7].

The management of the oxygen-inhibition layer has been proposed to optimize the surface properties of resin composites [8,9]. Consequently, many clinicians apply glycerin as an oxygen barrier during light curing to prevent atmospheric oxygen from interacting with free radicals at the material surface. This practice promotes a higher degree of conversion and improves the surface characteristics of the resin composite [9,10]. Nevertheless, various factors may affect the clinical longevity of restorations, and thus surface quality remains a concern. Final polishing is crucial for clinical success, yielding a smooth, glossy, and aesthetically pleasing surface [11].

Surface smoothness of a restoration not only influences aesthetics but also has functional implications, since a rough surface can promote bacterial plaque accumulation, staining, and recurrent caries [2,12,13]. This property is closely related to intrinsic material characteristics, such as the type of organic matrix and the size, composition, and distribution of filler particles [6,7,14].

Several studies have evaluated the impact of finishing and polishing procedures on the surface roughness of resin composites. However, debate persists in the literature regarding differences between dry and wet polishing methods [2,15]. Dry polishing has been associated with elevated temperatures generated during the procedure, which could compromise the structural integrity of the resin composite and alter its surface characteristics. In contrast, some studies report no significant differences in surface roughness between the two techniques [2,15,16]. Dry polishing offers improved visibility of the working area [17], while wet polishing may help dissipate heat generated by friction, potentially leading to a more uniform and sTable surface finish over time [2,18].

In this setting, it is pertinent to determine which polishing approach yields superior results for each resin composite type, aiming to optimize surface smoothness, durability, and aesthetics of restorations and thereby extend their service life [18,19]. Given the lack of consensus in existing findings [2,15], further research is warranted to compare these methods’ effects on resin composite surface properties. Therefore, the purpose of this *in vitro* study was to compare the surface roughness of three nanohybrid resin composites before and after dry and wet polishing. The null hypothesis was that no significant differences in surface roughness would be observed among the evaluated nanohybrid resin composites, before and after polishing, regardless of the method applied.

## Material and Methods

- Study Design

This experimental *in vitro* longitudinal study was conducted at the School of Stomatology of the Universidad Privada San Juan Bautista and at High Technology Laboratory Certificate (ISO/IEC Standard: 17025), Lima, Peru, between August and September 2024. Ethical approval was obtained from the Institutional Research Ethics Committee (No. 1338-2024-CIEI-UPSJB). The study followed the CRIS Guidelines (Checklist for Reporting In-vitro Studies) [20].

- Sample Size Calculation and Selection

**Figure 1:**
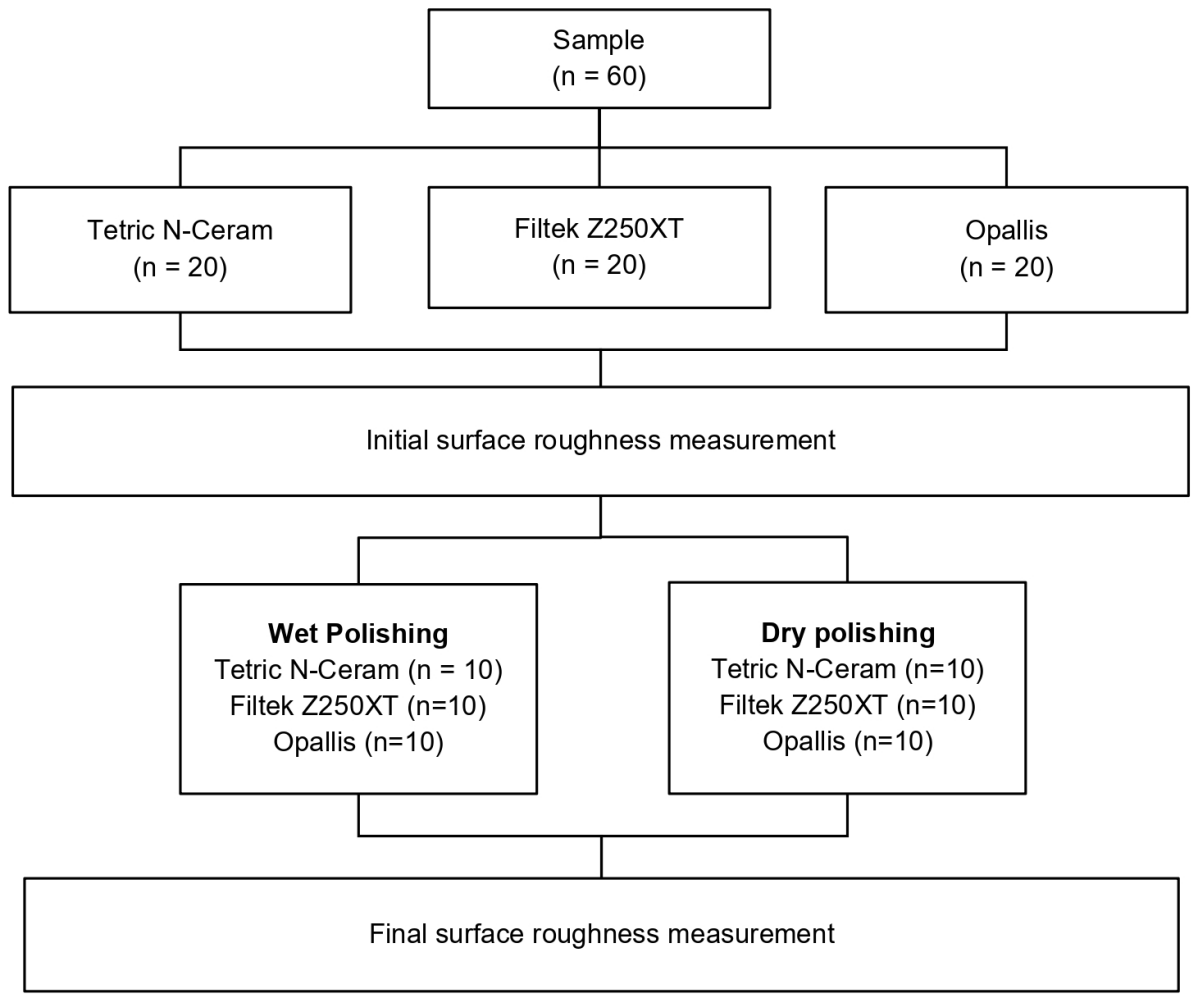
Random allocation of groups according to resin composite type and polishing technique.

The total sample size (n = 60) was determined based on data obtained from a prior pilot study in which five specimens were evaluated for each of the six experimental groups using a paired-measures design. The calculation was performed using the analysis of variance formula in G*Power statistical software (version 3.1.9.7), considering a significance level (α) of 0.05, a statistical power (1–β) of 0.80, and an estimated effect size of 0.97. Based on this, standardized discs were prepared for each resin composite (Tetric N-Ceram, Filtek Z250XT, and Opallis), yielding a total of 20 specimens per group (n = 20). These specimens were then randomly allocated into two subgroups (n = 10) according to the polishing technique used: wet or dry (Fig. 1).

- Sample Characteristics and Preparation

The resin composite discs were fabricated using a metallic mold measuring 6 mm in diameter × 4 mm in height [1,10,18]. The composites were placed in two 2-mm increments. On the final increment, glycerin (Alkofarma, Lima, Peru) was applied to the surface, followed by a celluloid strip and a 1-mm glass microscope slide, to ensure that the disc surfaces remained parallel [1,10]. The samples were then light-cured from the top using an Elipar DeepCure L LED curing unit (3M ESPE, Saint Paul, MN, USA) for 20 seconds at an intensity of 1470 mW/cm² [10]. The resin composites used were: Tetric N-Ceram (Ivoclar Vivadent, Schaan, Liechtenstein), Filtek Z250 XT (3M ESPE, St. Paul, MN, USA), and Opallis (FGM, Santa Catarina, Brazil) ( Table 1). After fabrication, all specimens were stored in distilled water at 37°C for 24 hours to allow for complete polymerization and post-curing shrinkage of the material [1,10-12]. Subsequently, baseline surface roughness was measured for all samples.

- Surface Roughness Measurement

Surface roughness was measured on all resin composite discs using a digital profilometer (Huatec SRT-6200®, China) with a resolution of 0.001 µm [1,10]. Three different areas on the surface of each specimen were evaluated, and the mean of these measurements was calculated to determine the absolute surface roughness (Ra) for each disc. Following the initial measurement, polishing was performed seven days later, and Ra was recorded again.

- Polishing techniques application

All finishing and polishing procedures were performed by the same operator [1,19]. These procedures were conducted using Sof-Lex discs (3M, ESPE, St. Paul, MN, USA), operated with an electric motor (EM-E6, W&H, Bürmoos, Austria) and a contra-angle handpiece (NSK, Tokyo, Japan) for 20 seconds per step, as per the manufacturer’s instructions, at a speed of 15,000 rpm using identical, unidirectional movements [1,17].

For the dry polishing groups, each specimen was polished for 20 seconds per disc, using a rotary motion and light, constant pressure. The discs were applied in decreasing order of grit size (from coarse to superfine) and replaced after each use. Between each disc change, the specimen was gently cleaned with soft tissue paper to remove debris [1,10].

In the wet polishing groups, the same protocol was followed, but with continuous water cooling during the procedure. Specimens were also rinsed with water for 5 seconds between each disc change [17]. After polishing, all specimens were stored in distilled water at 37 °C for 24 hours [10], after which the final absolute surface roughness was measured.

- Statistical Analysis

All data were imported into SPSS software (Statistical Package for the Social Sciences, version 24.0; IBM Corp., Armonk, NY, USA). Descriptive statistics were calculated using measures of central tendency and dispersion, including the mean and standard deviation. For inferential analysis, data normality was assessed using the Shapiro–Wilk test, and homoscedasticity was evaluated using Levene’s test. Based on these assumptions, the parametric Student’s t-test was used: the independent t-test to compare two groups and the paired t-test to compare two related measures. Statistical significance was set at *p* < 0.05.

## Results

When comparing the absolute surface roughness prior to dry or wet polishing, no significant differences were observed between the groups for each resin composite, indicating that the resin discs were properly standardized for Filtek Z250XT (*p* = 0.899), Opallis (*p* = 0.585), and Tetric N-Ceram (*p* = 0.721). Furthermore, no statistically significant differences were observed after dry or wet polishing for Filtek Z250XT (*p* = 0.066), Opallis (*p* = 0.124), or Tetric N-Ceram (*p* = 0.584) ( Table 2).

**Figure 2:**
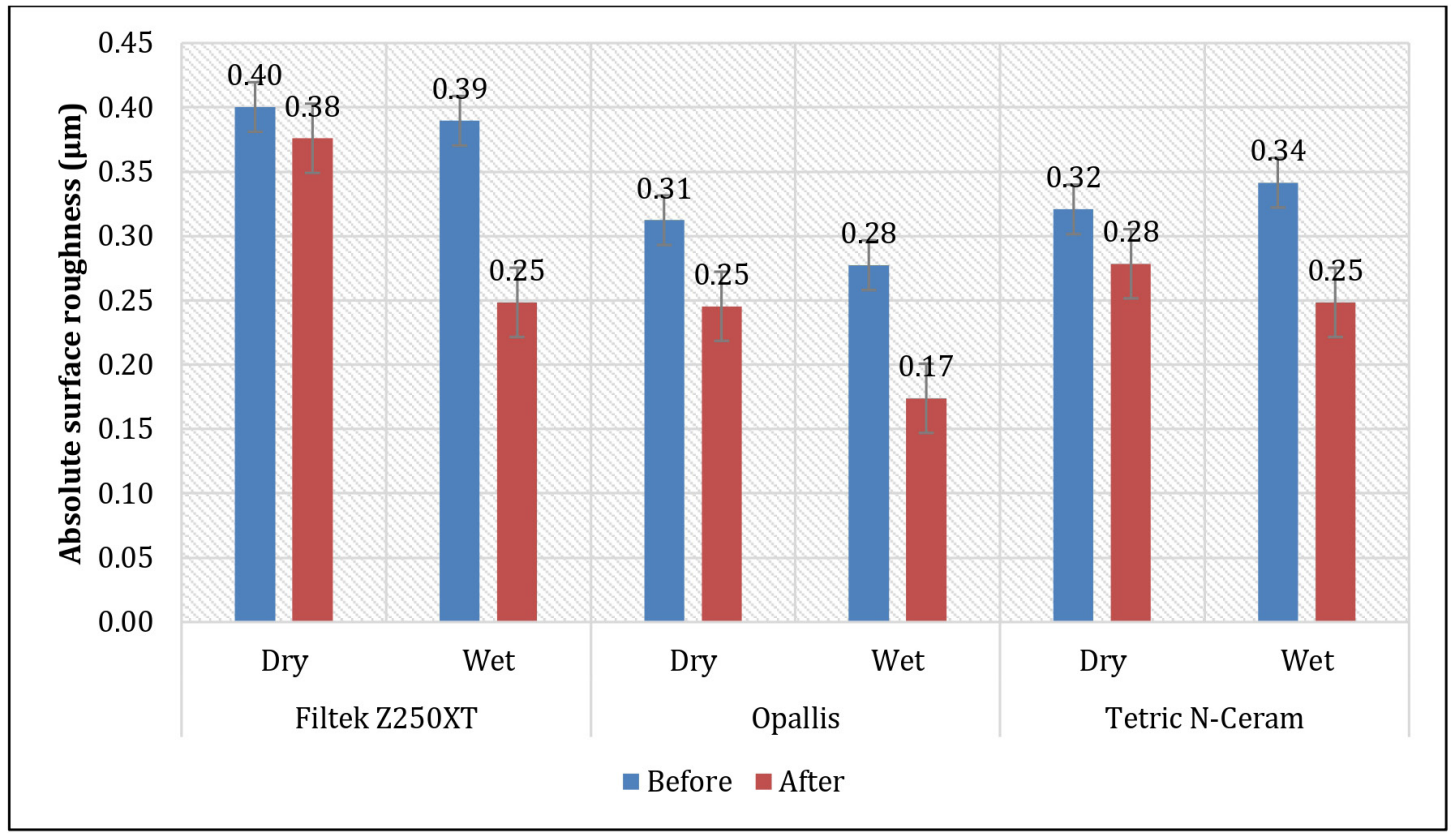
Mean absolute surface roughness (Ra) of three resin composites before and after dry or wet polishing.

When comparing the three resin composites before and after the application of dry or wet polishing, it was observed that Filtek Z250XT exhibited a significant decrease in Ra following wet polishing (*p* = 0.003), whereas no significant difference was found after dry polishing (*p* = 0.057). Moreover, Opallis showed a significant reduction in Ra after both dry (*p* = 0.044) and wet polishing (*p* < 0.001). Finally, Tetric N-Ceram also demonstrated a significant decrease in Ra after both dry (*p* = 0.001) and wet polishing (*p* < 0.001) ( Table 3, Fig. 2).

## Discussion

Since finishing and polishing are friction-based procedures, they generate heat that can potentially alter the physicochemical properties of resin composites. In this context, lubrication, such as water during wet polishing, has been proposed to dissipate heat and help preserve material integrity [2,12,13,17]. However, dry polishing offers practical advantages, including improved visibility of the working field, which supports its use in various situations. Therefore, the objective of this *in vitro* study was to compare the surface roughness of three nanohybrid resin composites before and after the application of dry and wet polishing techniques. Based on the results obtained, the null hypothesis was rejected.

The present study found that the resin composites Tetric-N Ceram and Opallis exhibited a reduction in absolute surface roughness with both polishing techniques, whereas Filtek Z250XT resin composite only showed improvement after wet polishing. These findings are consistent with those reported by Aydin *et al*. [15], who found no statistically significant differences between dry and wet polishing systems for their resin composite groups. However, they contrast with the results reported by Afifi *et al*. [18], De Freitas *et al*. [22], and Nasoohi *et al*. [23], who observed that dry polishing increased surface roughness in all evaluated groups, while wet polishing yielded significantly lower values. These discrepancies may be attributed to differences in the composition, type, and filler particle size of the resin composites used, which influence the material’s behavior during finishing and polishing [15]. In addition, the heat generated during dry polishing may degrade the bond between the matrix and filler, leading to particle separation and, consequently, an increase in surface roughness [23].

Regarding the resin composites evaluated, both Opallis and Tetric N-Ceram showed a significant reduction in their absolute surface roughness (Ra) following the application of both polishing techniques. One possible explanation lies in the smaller size of their filler particles, as Tetric N-Ceram contains particles ranging from 0.5 to 1.5 µm [10], while Opallis contains particles of approximately 0.5 µm [24]. This characteristic promotes a more homogeneous surface that is less prone to irregularities after polishing. In contrast, the Filtek Z250XT resin composite contains a combination of silica (20 nm) and a zirconia/silica blend with particle sizes ranging from 0.1 to 10 µm [25]. These spherical and variably sized particles tend to protrude more on the surface as the material wears, an effect that could be exacerbated by the heat generated during polishing [26].

Several studies have indicated that resin composites with smaller filler particles and reduced spacing between them allow for more efficient polishing, as they better protect the organic matrix from the effects of mechanical wear [10,26,27]. In this regard, the good performance of Opallis and Tetric N-Ceram, even under dry polishing conditions, could be attributed to their particle morphology, which facilitates a more uniform finish and, consequently, lower surface roughness [26].

The Filtek Z250XT resin composite showed a significant reduction in surface roughness when polished under wet conditions, whereas no improvement was observed with dry polishing. This difference may be attributed to its chemical composition, as it contains monomers such as Bis-GMA, TEGDMA, and UDMA, whose hydrophilic functional groups promote water absorption within the polymer network [28]. This characteristic increases the matrix’s susceptibility to hydrolytic and thermal degradation, potentially compromising the filler–matrix interface and deteriorating the surface when dry polishing is applied. Moreover, the heat generated during this procedure may exacerbate such degradation, favoring filler debonding and a subsequent increase in surface roughness [17,23,29]. It has also been reported that abrasive particles from the Sof-Lex system may become embedded in the resin composite surface under these conditions [22]. In contrast, the use of polishing with irrigation could effectively dissipate the frictional heat generated, thereby protecting matrix integrity and explaining the observed improvement in surface properties. Unlike Filtek Z250XT, resin composites such as Opallis and Tetric N-Ceram contain Bis-EMA, a more hydrophobic monomer associated with lower water sorption and solubility [28,30,31], which may confer greater stability against the effects of dry polishing.

It has been reported that bacterial accumulation increases significantly when the surface roughness of resin composites exceeds 0.2 µm, while patients do not perceive irregularities when roughness remains below 0.3 µm [23]. In the present study, all evaluated resin composites exhibited accepTable surface roughness values after the polishing procedure, with the exception of Filtek Z250XT subjected to dry polishing, which reached an average value of 0.38 µm, exceeding both clinically relevant thresholds.

Although this was an *in vitro* study, the findings provide useful insights that may help guide clinical decision-making. Surface finishing techniques should be selected with consideration of the resin composite’s chemical composition and physical properties to achieve optimal restorative outcomes. A smoother surface may enhance aesthetic results and reduce plaque accumulation. However, due to the limitations inherent in laboratory conditions, further *in vivo* or clinical studies are needed to confirm these observations. Additionally, the lack of transparency regarding the filler composition and monomer content in commercial composites may contribute to variability in polishing performance [30,32].

Among the limitations of the present study, it should be noted that, as an *in vitro* investigation, the results cannot be directly extrapolated to the clinical setting, where factors such as the presence of saliva, enzymes, food intake, and functional wear may influence surface roughness [1,10]. In addition, the evaluation focused on flat, buccal surfaces; therefore, the findings may not adequately represent clinical outcomes on occlusal surfaces, where anatomical irregularities hinder complete access during polishing procedures [1].

As a methodological strength, all specimens were standardized, ensuring homogeneous baseline conditions across the different resin composite groups. The Sof-Lex polishing system was also employed, which is well-documented for providing a smooth surface finish with high gloss, minimizing the formation of grooves and irregularities that may promote plaque accumulation and extrinsic staining [1,10,27]. Another relevant aspect was the seven-day waiting period prior to polishing. This decision was based on evidence indicating that hygroscopic expansion of the material occurs during this time, thereby reducing the risk of microcracks associated with premature polishing [23]. Furthermore, the application of glycerin on the final resin layer during light curing to inhibit the oxygen-inhibition layer ensured optimal polymerization and surface quality of the resin composites [10]. It has also been reported that resin composite polymerization reaches only about 75% conversion within the first 10 minutes, so immediate polishing may induce plastic deformation of the restored surface [28,29].

It is recommended to evaluate color stability as an additional variable, due to its close relationship with the polishing system, considering its impact on the durability, esthetic appearance, and functional integrity of the restoration. Likewise, it is suggested to incorporate thermocycling protocols to simulate intraoral thermal variations and to compare the performance of other finishing and polishing systems beyond the Sof-Lex discs [27].

## Conclusions

Both dry and wet polishing are effective in reducing the surface roughness of Opallis and Tetric N-Ceram resin composites, whereas in Filtek Z250XT resin composite, this reduction was significant only with wet polishing. These findings suggest that the effectiveness of the polishing procedure may vary according to the type of resin composite, which should be considered when selecting clinical finishing and polishing protocols to optimize the surface properties of restorative materials.

## Figures and Tables

**Table 1:** Table Technical profile of the materials used.

Product	Composition	Filler % (wt-vol)	Manufacturer	Lot
Filtek 250 XT	Matrix: BIS-GMA, TEGDMA, UDMA Filler: Silane treated ceramic, Bisphenol a polyethylene glycol diether dimethacrylate	82 wt 68 vol	3M, ESPE, St. Paul, MN, USA.	10238336 10238336
Tetric N-Ceram	Matrix: Bis-GMA, Bis-EMA, UDMA Filler: Dimethacrylates, additives, catalysts, stabilizer sand pigments, barium glass, ytterbium trifluoride, mixed oxide and prepolymerized filler	81 wt 57 vol	Ivoclar Vivadent, Schaan, Liechtenstein.	Z06LMY Z06FOR
Opallis	Matrix: Bis-GMA, Bis-EMA, UDMA, TEGDMA. Filler: The loads are a combination of silanized barium-aluminum silicate glass and nanoparticles of silicon dioxide, camphorquinone as photoinitiator, accelerators, stabilizers and pigments	79.8 wt 58 vol	FGM, Santa Catarina, Brazil.	060122 060122
Sof-Lex System	Aluminum oxide abrasive discs	-----------	3M, ESPE, St. Paul, MN, USA.	NE04288 NC93140 NC80025 NE04500

**Table 2:** Table Descriptive values and comparison of the absolute surface roughness (Ra) of three resin composites before and after dry or wet polishing.

Resin composite	Polishing	n	Before polishing					After polishing				
			Mean	SD	95% CI		p*	Mean	SD	95% CI		p*
					LL	UL				LL	UL	
Filtek Z250XT	Dry	10	0.40	0.17	0.28	0.53	0.899	0.38	0.17	0.25	0.50	0.066
	Wet	10	0.39	0.20	0.25	0.53		0.25	0.12	0.16	0.33	
Opallis	Dry	10	0.31	0.12	0.22	0.40	0.585	0.25	0.06	0.20	0.29	0.124
	Wet	10	0.28	0.15	0.17	0.39		0.17	0.13	0.08	0.26	
Tetric N-Ceram	Dry	10	0.32	0.14	0.22	0.42	0.721	0.28	0.12	0.19	0.37	0.584
	Wet	10	0.34	0.12	0.26	0.43		0.25	0.12	0.16	0.33	

n: sample size; SD: standard deviation; 95% CI: 95% confidence interval; LL: lower limit; UL: upper limit. *Based on Student’s t-test for independent samples (*p* < 0.05).

**Table 3:** Table Comparison of absolute surface roughness (Ra) of three resin composites before and after the application of dry or wet polishing.

Resin composite	Polishing	f -i	SD	95% CI		t	p*
				UL	LS		
Filtek Z250XT	Dry	-0.02	0.04	-0.05	0.00	-2.18	0.057
	Wet	-0.14	0.11	-0.22	-0.06	-3.97	0.003*
Opallis	Dry	-0.07	0.09	-0.13	0.00	-2.34	0.044*
	Wet	-0.10	0.05	-0.14	-0.07	-6.19	<0.001*
Tetric N-Ceram	Dry	-0.04	0.03	-0.06	-0.02	-5.08	0.001*
	Wet	-0.09	0.02	-0.10	-0.08	-17.85	<0.001*

*Based on the paired Student’s t-test (*p* <0.05, statistically significant differences);(Ⴟf– Ⴟi): Mean difference; (Ⴟf): After polishing; (Ⴟi): Before polishing; SD: Standard deviation; 95% CI: 95% confidence interval; LL: Lower limit; UL: Upper limit; t: Student’s t-test.

## Data Availability

The datasets used and/or analyzed during the current study are available from the corresponding author.

## References

[1] Carrillo-Marcos A, Salazar-Correa G, Castro-Ramirez L, Ladera-Castañeda M, López-Gurreonero C, Cachay-Criado H (2022). The microhardness and surface roughness assessment of bulk-fill resin composites treated with and without the application of an oxygen-inhibited layer and a polishing system: An in vitro study. Polymers (Basel).

[2] Silva JP, Coelho A, Paula A, Amaro I, Saraiva J, Ferreira MM (2021). The Influence of Irrigation during the Finishing and Polishing of Composite Resin Restorations-A Systematic Review of In Vitro Studies. Materials (Basel).

[3] Ramírez L, Colán PR, Valencia JJ, Guevara JO, Morales R (2022). Does glycerin influence the color stability of composite resin?. Rev Cubana Estomatol.

[4] Gutarra Vargas J, Ulloa Cueva T, Espinoza Salcedo M (2022). Surface hardness of a Bulk Fill resin according to polishing time. Odontol Activa Rev Científica.

[5] Kalita T, Kalita C, Das L, Kataki R, Boruah LC, R A (2023). Comparative evaluation of colour stability and surface roughness of nanohybrid composite resins in mouth rinse and colouring beverages. Cureus.

[6] Chowdhury D, Mazumdar P, Desai P, Datta P (2020). Comparative evaluation of surface roughness and color stability of nanohybrid composite resin after periodic exposure to tea, coffee, and Coca-Cola” An in vitro profilometric and image analysis study. J Conserv Dent.

[7] Szczepaniak ME, Krasowski M, Bołtacz-Rzepkowska E (2022). The effect of various polishing systems on the surface roughness of two resin composites-an in vitro study. Coatings.

[8] Ozan G, Mert M, Tugce A, Yildirim Z, Yucel Y (2021). A Comparison Of Surface Roughness Values of Various Restorative Materials Immersed in Pedodontic Pre-and Probiotics. Biointerface Research in Applied Chemistry.

[9] Panchal A, Asthana G (2020). Oxygen inhibition layer: A dilemma to be solved. J Conserv Dent [Internet].

[10] Gaviria-Martinez A, Castro-Ramirez L, Ladera-Castañeda M, Cervantes-Ganoza L, Cachay-Criado H, Alvino-Vales M (2022). Surface roughness and oxygen inhibited layer control in bulk-fill and conventional nanohybrid resin composites with and without polishing: in vitro study. BMC Oral Health.

[11] Vaz BAS, Lima RBW e, Andrade AKM de, Meireles SS, Silva FDSCM e, Barbosa LMM (2021). Evaluation of surface roughness of restorative dental composites: influence of depth cure, time and storage medium. RSD.

[12] Aydın N, Topçu F, Karaoğlanoğlu S, Oktay E, Erdemir U (2021). Effect of finishing and polishing systems on the surface roughness and color change of composite resins. J Clin Exp Dent.

[13] Şivgan Güner Z, Bolgül B, İnandı T (2021). Evaluation of the color stability and surface roughness of dual-cure, bulk-fill composites. Int Dent Res.

[14] Oliveira LPS, Gomes MA, da Silva JHR, Silva CC, Pontes DG, Regalado DF (2021). The effect of three polishing systems on the surface rugosity of one composite resin. Dent Oral Biol Craniofac Res.

[15] Aydın N, Karaoğlanoğlu S, Kılıçarslan MA, Oktay EA, Ersöz B (2022). Effect of wet and dry polishing conditions by two finishing and polishing systems on the surface roughness and color changes of two composite resin restoratives: an in vitro comparative study. J Adv Oral Res.

[16] Bayraktar D, Doğan D, Ercan D (2013). Effect of the different polishing systems and techniques on surface roughness of three different composite resin. J Dent Fac Atatürk Uni.

[17] Ghasemi A, Mohammadzadeh A, Molaei M, Sheikh-Al-Eslamian SM, Karimi M (2023). Effect of wet and dry finishing and polishing technique on microhardness and flexural strength of nanocomposite resins. Int J Dent.

[18] Afifi R, Aly S (2019). Effect of Wet and Dry Finishing and Polishing on Surface Roughness and Microhardness of Bulk fill Resin Composites. Egyptian Dental Journal.

[19] Aljamhan A, Habib SR, AlSarhan MA, AlZahrani B, AlOtaibi H, AlSunaidi N (2021). Effect of finishing and polishing on the surface roughness of Bulk fill composites. Open Dent J.

[20] Krithikadatta J, Gopikrishna V, Datta M (2014). CRIS Guidelines (Checklist for Reporting In-vitro Studies): A concept note on the need for standardized guidelines for improving quality and transparency in reporting in-vitro studies in experimental dental research. J Conserv Dent.

[21] Okida R, Hoshino I, Romanini L, Fontes A, Esteves L, Anchieta R (21). Influence of different polishing and aging periods on the surface roughness of composite resins. Research, Society and Development.

[22] De Freitas M, de Freitas D, de Almeida L, Magalhães A, Cardoso P, Decurcio R (2019). Influence of wet finishing and polishing on composite resins: Surface roughness, color stability and surface morphology. Rev Odontológica Bras Cent.

[23] Nasoohi N, Hoorizad M, Tabatabaei SF (2017). Effects of wet and dry finishing and polishing on surface roughness and microhardness of composite resins. J Dent (Tehran).

[24] Valinoti AC, Neves BG, da Silva EM, Maia LC (2008). Surface degradation of composite resins by acidic medicines and pH-cycling. J Appl Oral Sci.

[25] Shah SS, Patel NK, Yagnik KP, Vyas A, Doshi P, Keshrani PR (2024). Comparative evaluation of microhardness of three restorative materials after immersion in chlorhexidine mouthwash: an in vitro study. J Conserv Dent Endod.

[26] Ferretti MA, Pereira R, Lins RBE, Soares MGC, Pinto LJH, Martins LRM (2021). Characterization of low-cost Brazilian resin composites submitted to tooth brushing. Braz Oral Res.

[27] Ramírez-Vargas GG, Ladera-Castañeda MI, López-Gurreonero C, Cornejo-Pinto A, Cachay-Criado H, Cervantes-Ganoza LA (2022). Surface Roughness in Nanoparticle Resin Composites Subjected to Two Polishing Systems: An In vitro Comparative Study. J Int Soc Prev Community Dent.

[28] Szczesio-Wlodarczyk A, Domarecka M, Kopacz K, Sokolowski J, Bociong K (2021). An Evaluation of the Properties of Urethane Dimethacrylate-Based Dental Resins. Materials.

[29] Kaminedi RR, Penumatsa NV, Priya T, Baroudi K (2014). The influence of finishing/polishing time and cooling system on surface roughness and microhardness of two different types of composite resin restorations. J Int Soc Prev Community Dent.

[30] Díaz Benítez MG, Echeverría Escobar SA, Talavera Ovelar LA, Bañuelos-Gómez MA, Michel de Román T, Meza JG (2023). Evaluación in vitro de la absorción de agua y variación de color de resinas compuestas sumergidas en dos sustancias pigmentantes. Rev Fac Odontol (Córdoba).

[31] Agarwal D, Rishi R, Subhasis S, Prakash P (2015). Comparative evaluation of the effect of water on three different light cured composite restorative materials: an in vitro study. Scholars Academic Journal of Biosciences.

[32] Molaei M, Mohammadzadeh A, Ghasemi A, Badiee M (2024). Effect of dry and wet finishing and polishing on color change and opacity of nanofill and nanohybrid composites. BMC Oral Health.

